# Commotio Cordis

**Published:** 2007-10-22

**Authors:** Christopher Madias, Barry J Maron, Alawi A Alsheikh-Ali, N A Mark Estes III, Mark S Link

**Affiliations:** 1 Cardiac Arrhythmia Center, Division of Cardiology, Tufts-New England Medical Center, Boston, Massachusetts, USA; 2 Minneapolis Heart Institute Foundation, Minneapolis, Minnesota, USA

**Keywords:** Commotio cordis, Ventricular Fibrillation, Athletes, Sudden Death, Mechano-electric coupling

## Abstract

Sudden arrhythmic death as a result of a blunt chest wall blow has been termed Commotio Cordis (CC). CC is being reported with increasing frequency with more than 180 cases now described in the United States Commotio Cordis Registry. The clinical spectrum is diverse; however young athletes tend to be most at risk, with victims commonly being struck by projectiles regarded as standard implements of the sport. Sudden death is instantaneous and victims are most often found in ventricular fibrillation (VF). Chest blows are not of sufficient magnitude to cause any significant damage to overlying thoracic structures and autopsy is notable for the absence of any structural cardiac injury. Development of an experimental model has allowed for substantial insights into the underlying mechanisms of sudden death. In anesthetized juvenile swine, induction of VF is instantaneous following chest impacts that occur during a vulnerable window before the T wave peak. Other critical variables, including the impact velocity and location, and the hardness of the impact object have also been identified. Rapid left ventricular pressure rise following chest impact likely results in activation of ion channels via mechano-electric coupling. The generation of inward current through mechano-sensitive ion channels results in augmentation of repolarization and non-uniform myocardial activation, and is the cause of premature ventricular depolarizations that are triggers of VF in CC. Currently available chest protectors commonly used in sport are not adequately designed to prevent CC. The development of more effective chest protectors and the widespread availability of automated external defibrillators at youth sporting events could improve the safety of young athletes.

## Introduction

Sudden cardiac death as a result of a blunt and often innocent-appearing chest wall blow has been termed commotio cordis (CC). Derived from Latin and meaning *disturbance of the heart*, it is now apparent that CC is a primary electrical event, with the instantaneous induction of ventricular fibrillation (VF) resulting from non-penetrating chest wall impacts that are usually not of sufficient force to cause any significant structural injury to the heart [1,2]. This characteristic differentiates CC from *contusio cordis*, in which high impact blows result in direct myocardial tissue damage, often associated with injury to the overlying structures of the chest and thorax. Although the clinical context has proven diverse, many instances of CC occur as a result of chest impact with projectiles used in organized sports, such as baseball, hockey, and lacrosse3. In the United States, CC has now been recognized as a leading cause of sudden death in youth athletics and is being reported with increasing frequency. Over the last decade, CC has achieved broader visibility through a series of reports detailing its clinical spectrum [3,4]. At the same time, the development of a contemporary experimental model has shed substantial light into the pathophyisology [1]. This article will review the clinical spectrum of CC, address recent insights into the underlying mechanisms, and discuss strategies to prevent this tragic event.

## Clinical spectrum

Since being initiated in 1996, the United States Commotio Cordis Registry (USCCR - Minneapolis, Minnesota) has now accrued more than 180 cases [3-5]. As awareness of this phenomenon grows, CC is being reported with increasing frequency, with most cases in the registry (75%) clustered from the years 1988 to present [6]. However, the actual incidence remains unknown as many cases are still likely missed due to continued lack of recognition and underreporting. CC has most commonly been described in the setting of organized sport (Table 1), with most victims having been struck in the chest by standard projectiles used in the game [3]. Generally, projectiles that result in CC have a dense solid core, such as a baseball, hockey puck, or lacrosse ball. Only 2 cases in the USCCR have been attributed to impact with a cricket ball. However, this low incidence likely reflects the relative lack of popularity of cricket in the US and the fact that chest impact in cricket is a rare event. Projectiles with a non-solid core tend to collapse on contact and absorb much of the impact energy. Only a single event has been attributed to chest impact with an air-filled soccer ball. In almost all cases, chest impacts that resulted in CC occurred to the left of the sternum, directly over the cardiac silhouette. Estimated velocities of pitched baseballs were 48 to 80 km/h (30-50 mph). Interestingly, 38% of the individuals competing in organized sports were wearing standard commercially available chest wall protection at the time of their event [7]. However, in 25 of these 32 cases, the chest wall protector did not adequately cover the left chest or precordium at the time of impact.

**Figure 1:**
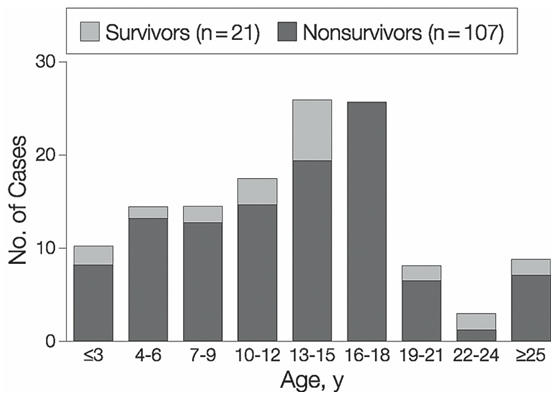
Age at the time of event in the United States Commotio Cordis Registry. Reprinted with permission from: Maron BJ, Gohman TE, Kyle SB, Estes NA, 3rd, Link MS. Clinical profile and spectrum of commotio cordis. JAMA 2002;287(9):1142-6. Copyright ©  (2002), American Medical Association. All rights reserved.

Although commonly associated with sport, CC has now been reported in a diverse spectrum of non-sports related activities [3]. Many of these cases occurred in association with happenings of everyday life that resulted in often unintentional and innocuous-appearing chest blows. Some such examples include a 23 year old man fatally striking his friend in the chest as a mutually agreed upon remedy for hiccups. In two other cases, a 2-year old girl was incidentally struck in the chest by the head of her pet dog and a 5-year old boy died instantly after being struck in the chest by a circular plastic sledding saucer. Young males (median age of 14 years) appear to be most at risk from CC [3]. This susceptibility has been partially attributed to the compliant chest walls of children that allow for greater transmission of impact energy to the myocardium. Only 28% of the cases in the USCCR were aged over 18 years, with the oldest victim a 44 year-old woman (Figure 1).

The overall survival rate in known victims of CC is only 15%, with successful resuscitation often quite difficult [3,8]. Initial ECG data (recorded in the emergency room or by emergency medical technicians in the field) was available in 82 patients in the USCCR. Analysis revealed 33 cases of VF, 3 with ventricular tachycardia, 3 with bradyarrhythmias, 2 with idioventricular rhythm, and 1 with complete heart block. Forty of the cases documented asystole, which was unlikely to be the initial rhythm after impact, and is more likely a result of prolonged time from event to rhythm documentation. Application of early resuscitation and defibrillation appears to be the most important determinant of survival, as with other causes of VF. Cardiopulmonary resuscitation was known to have been performed in 106 of the individuals in the USCCR [3]. Of 68 cases in which early resuscitation was instituted (< 3 minutes), 17 survived (25%). In the cases where resuscitation was substantially delayed (> 3 minutes) only 1 out of 38 survived (3%).

## Pathophysiology

**Figure 2:**
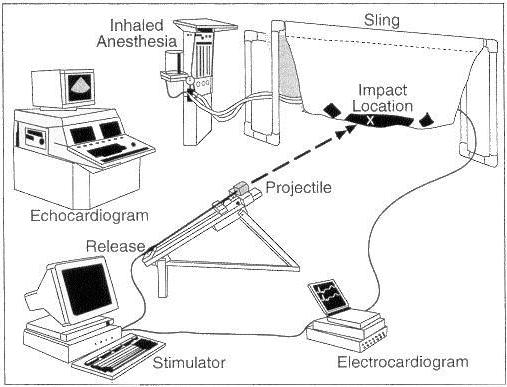
Laboratory and study design for the commotio cordis model. An anesthetized and intubated animal is positioned prone in a sling. Under echocardiographic guidance, a ball affixed to an aluminum shaft is impacted on the chest directly over the base of the left ventricle. An electrophysiologic stimulator triggered from the surface electrocardiogram is used to gate impacts to the cardiac cycle. A chronograph measures the impact velocity. Reprinted with permission from: Link MS, Wang PJ, VanderBrink BA, et al. Selective activation of the K(+)(ATP) channel is a mechanism by which sudden death is produced by low-energy chest-wall impact (Commotio cordis). Circulation 1999;100(4):413-8. Copyright ©  (1999), The American Heart Association.

**Figure 3:**
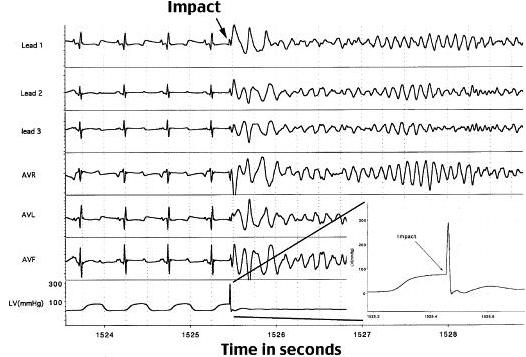
Six lead electrocardiogram and intraventricular pressure measurement from and 11 kg swine undergoing a 48 km/h (30 mph) chest wall impact with an object the shape and weight of a standard baseball. Ventricular fibrillation is produced immediately upon impact within the vulnerable zone of repolarization (10-30 ms prior to the peak of the T-wave). Reprinted from: Link MS, Maron BJ, VanderBrink BA, et al. Impact directly over the cardiac silhouette is necessary to produce ventricular fibrillation in an experimental model of commotio cordis. J Am Coll Cardiol 2001;37(2):649-54. Copyright 2001, with permission from Elsevier.

It is now apparent that CC is a primary electrical event, with sudden death resulting from the instantaneous induction of VF initiated by a chest wall blow. Autopsy data generally has not revealed any underlying congential or acquired heart disease in victims. Available evidence suggests that the pathophysiology of impact-induced VF is multi-factorial and requires the precise confluence of several variables. Development of an experimental animal model has allowed for a deeper understanding of the underlying mechanisms. This model attempts to mimic the clinical profile of CC, and entails propelling projectiles commonly used in sport (baseballs and lacrosse balls) at the chest wall of anesthetized juvenile swine [1]. Release and subsequent impact of the balls are gated to the cardiac cycle by a cardiac stimulator with triggering from the surface electrocardiogram of the swine (Figure 2). Initial experiments involving this animal model defined a narrow window of vulnerability within the cardiac cycle that is critical for the development of CC. When impacts occurred precisely within 10 to 30 milliseconds before the peak of the T wave, VF was consistently produced (Figure 3). VF was instantaneous and was not preceded by premature ventricular contractions (PVC), ST-segment changes, or heart block. Chest impacts occurring in other portions of the cardiac cycle produced various other electrophysiologic effects - including ST-segment elevation, PVC, transient heart block, and left bundle branch block - but never resulted in VF.

**Figure 4:**
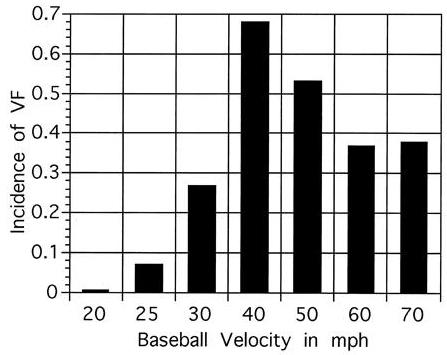
Incidence of ventricular fibrillation (VF) induced by chest wall impacts at the vulnerable period of repolarization (10-30 ms prior to the peak of the T-wave) with a regulation baseball propelled at velocities ranging from 32 to 113 km/h (20 to 70 mph) in the swine model of commotio cordis. The incidence of VF relative to the velocity of chest impact exhibited a Gaussian distribution with peak incidence at 64 km/h (40 mph) (p < 0.0001 by logistic regression). Reprinted from: Link MS, Maron BJ, Wang PJ, VanderBrink BA, Zhu W, Estes NA, 3rd. Upper and lower limits of vulnerability to sudden arrhythmic death with chest-wall impact (commotio cordis). J Am Coll Cardiol 2003;41(1):99-104. Copyright 2003, with permission from Elsevier.

Several other factors have also been identified as crucial to the development of VF in this model of CC. Using echocardiographic guidance, the importance of impact location directly over the anatomic position of the heart was revealed [9]. VF occurred most commonly with blows directly over the center of the cardiac silhouette (30% of impacts) versus those over the left ventricular base (13% of impacts) or apex (4% of impacts). Thoracic impacts not overlaying the heart did not result in VF or other electrophysiologic effects. In addition, a relationship between the hardness of the impact object and the likelihood of inducing VF was also identified. Impacts with softer safety baseballs were associated with a lower incidence of VF than impacts with harder standard balls [10]. Finally, the importance of the velocity of chest wall impacts was also systematically evaluated [11]. Baseballs were propelled with velocities ranging from 32 to 113 km/h (20 to 70 mph) and timed to impact on the vulnerable 20 ms window on the upstroke of the T wave. The plot of impact velocity relative to incidence of VF exhibited a Gaussian distribution. The threshold velocity to cause VF was 40 to 48 km/h (25 to 30 mph) and as impact velocity increased, the incidence of VF rose to a peak of nearly 70% of impacts at 64 km/h (40 mph). At velocities > 80 km/h (50 mph), however, the likelihood of VF decreased (Figure 4). This observation is consistent with the observed clinical scenario of CC in youth baseball, where baseball velocities are estimated to range between 48 to 80 km/h (30 to 50 mph).

**Figure 5:**
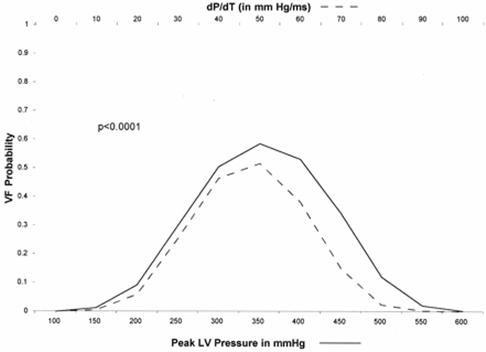
The probability of ventricular fibrillation (VF) relative to the peak left ventricular (LV) pressure and LV pressure over time (dP/dt) in 8-12 kg swine undergoing 48 km/h (30 mph) chest wall impacts with a baseball. The data exhibit a Gaussian distribution (p < 0.0001 by logistic regression). The highest incidence of VF was evident with peak LV pressures between 250 and 450 mmHg. Reprinted from: Link MS, Maron BJ, VanderBrink BA, et al. Impact directly over the cardiac silhouette is necessary to produce ventricular fibrillation in an experimental model of commotio cordis. J Am Coll Cardiol 2001;37(2):649-54. Copyright 2001, with permission from Elsevier.

The importance of the location, hardness, and velocity of chest wall impacts in CC relate to the effects of these variables on induction of a critical left ventricular (LV) pressure that is necessary to produce VF. In experiments of impact velocity, higher velocities correlated with the generation of greater peak instantaneous LV pressures. As with impact velocities, the risk of VF correlated with the LV pressure rise created by the chest wall blow in a Gaussian distribution (Figure 5) [8,11,12]. The highest incidence was evident with peak LV pressures of 250 to 450 mmHg and decreased with pressures above and below this range. Thus, these data suggest that there is a lower and upper limit of vulnerability of LV pressures resulting in VF and that the instantaneous LV pressure rise produced by the chest blow mediates the electrophysiologic consequences of CC.

Mechanical stimulation of the myocardium resulting in electrical events is well-described, occurring in such circumstances as catheter induced ectopic beats and thumping of the chest wall during asystole to produce PVCs [5,8]. This phenomenon, termed mechano-electric coupling, has been attributed to the presence of mechano-sensitive ion channels that are activated by deformation of the myocardial cell membrane. In CC, rapid rise of ventricular pressure immediately following chest impact results in VF mediated through resultant myocardial stretch and the activation of ion channels. CC appears to share certain electrical similarities with myocardial ischemia, including ST-segment elevation and the phenomenon of R on T causing VF [13,14]. Activation of the K^+^_ATP_ channel is primarily responsible for ST-segment elevation noted in myocardial infarction, and contributes to the increased risk of VF associated with ischemia. In addition, mechano-sensitivity of the K^+^_ATP_ channel has been previously demonstrated in a rat model [15]. In our model of CC, infusion of glibenclamide, a K^+^_ATP_ channel inhibitor, reduced the magnitude of ST-elevation and the incidence of VF following chest blows [13]. Our results suggest that the immediate activation of the mechano-sensitive K^+^_ATP_ channel by chest wall impacts is in part responsible for the induction of VF in CC. Other stretch-sensitive ion channels are also likely to be involved. Interestingly, however, blockade of the non-selective cation stretch-activated channel (SAC) with streptomycin did not prevent induction of VF in our model [12].

In CC, the inward current generated through the opening of mechano-sensitive ion channels results in ventricular depolarizations that in turn, trigger development of VF. However, ventricular depolarization alone is not sufficient to result in reentrant arrhythmia that underlies the mechanism of VF. Thus, initiation of VF in CC appears to require at least two features: (i) a *trigger* - premature ventricular depolarization - occurring in the setting of (ii) a *susceptible myocardial substrate* [5]. The necessity of both trigger and substrate is illustrated in experiments of impact velocity [8]. PVCs were observed in nearly 70% of impacts that did not result in VF. Thus, a trigger (ventricular depolarization) was produced, but did not result in VF, presumably due to the absence of appropriate substrate.

Interestingly, both trigger for CC and the susceptible myocardial substrate are in part created by a chest wall blow occurring in the vulnerable portion of the cardiac cycle. Susceptibility to development of CC relates to dispersion of repolarization that is present during the vulnerable period of the cardiac cycle when chest impact occurs. Recent data by Bode et al support this hypothesis [16]. Fluid-filled balloons were placed in the LV of Langendorff perfused rabbit hearts and increasing volume and pressure pulses were applied at different points of the cardiac cycle. VF was induced only when balloon inflation occurred within a vulnerable window of 35ms to 88ms after the initiation of an action potential. This vulnerable window corresponded to the time of spontaneous increase in repolarization dispersion. Even more interesting was the observation that as compared to baseline, pressure pulses that induced VF resulted in a * further increase* in repolarization dispersion. Thus, it appears that the upstroke of the T wave signifies a window of *potential* vulnerability for development of VF in CC, due to spontaneous increase in repolarization dispersion. The potential vulnerability for the induction of VF is *realized* when chest impact results in sudden elevation in LV pressure leading to further increase in repolarization dispersion. Analogous to this hypothesis is the R on T phenomenon. In non-ischemic myocardium, premature ventricular depolarization during the T wave does not normally induce VF. Thus, continuous ventricular pacing (VOO) is generally safe. However, with the increase in repolarization dispersion in the setting of ischemia, the potential for inducing VF can be realized when a PVC falls on the vulnerable portion of the T wave [17,18].

In addition, the experiments by Bode et al provide further insight into the electrical properties by which increase in repolarization dispersion might produce VF in CC [16]. In their model, it was observed that the LV myocardium was not excited simultaneously by a global pressure pulse. Instead it was noted that the earliest activation occurred at the LV site with the shortest repolarization time and occurred considerably later at sites with longer repolarization times. VF was induced when a sufficient electrical gradient was able to activate myocardium with early local recovery, but failed to activate myocardium that was refractory. Based on these findings, non-uniform excitation might thus form the basis for the initiation of reentry and the induction of VF in CC.

Although activation of the K^+^_ATP_ channel is shared by both CC and ischemic myocardium, the mechanism of activation is quite different. In our model, angiography performed immediately after impact in animals that developed VF did not reveal any evidence of stenosis or spasm in epicardial coronary arteries [1]. Myocardial perfusion imaging with technetium 99m sestamibi performed after impact revealed only small mild apical defects in a minority (25%) of the animals tested. In addition, left ventriculograms and echocardiograms performed immediately after defibrillation revealed only mild apical or distal septal hypokinesis, regions distant from the area of precordial impact [1]. On pathologic examination, structural cardiac damage has not been observed with impact velocities of less than 80 km/h (50 mph) [8].

## Prevention and Therapy

Deaths of young athletes from CC are tragic and often highly visible events. In the United States, several preventive strategies have been considered, including increasing the use of softer balls and chest wall protectors in organized youth sports. Safety baseballs with soft rubber cores have been designed to reduce the risk of head and other bodily injury from traumatic impacts. In our experimental model, safety baseballs significantly reduced, but did not eliminate, the risk of sudden cardiac death with chest impacts at 48 km/h and 64 km/h (30 and 40 mph) [10]. Despite these data, widespread use of safety baseballs in youth sports has yet to be adopted.

In the United States, chest protectors are marketed with claims of protecting athletes from chest wall trauma. However, in the USCCR, more than 38% of the fatal impacts occurring during organized sport involved individuals who were wearing a chest protector [3,7]. In several of these cases, migration of the protector exposed the chest to a direct impact. In other cases, however, projectiles were known to strike on the chest protector, which failed to prevent the fatal CC event. Recently, the effectiveness of commercially available lacrosse and baseball chest protectors was assessed for the prevention of sudden cardiac death [19]. None of the currently available chest protectors was shown to significantly decrease the incidence of VF when compared to controls. In accord with our previous findings, peak LV pressure produced by chest wall impacts correlated linearly with the probability of VF in these experiments. Using a 3 rib biomechanical dummy model, Viano et al also demonstrated the ineffectiveness of several commercial chest protectors in reducing the potential risk of CC [20]. The majority of the chest protectors evaluated in these studies were composed of a compliant layer(s) of closed cell foam of varying thickness and density that is intended to dissipate the energy of an impact. The ineffectiveness of this design in reducing the incidence of VF is likely due to its inability to adequately reduce the peak LV pressure generated by chest wall impact. It is clear that further research on the development of an adequate chest protector for the prevention of CC is needed.

Likely due to a lack of early recognition and the failure to initiate timely aggressive resuscitation and defibrillation, the survival rate in the USCCR is only about 15% [3]. Survival is most likely to occur with the institution of cardiopulmonary resuscitation and defibrillation within 3 minutes of the incident event. Similar outcomes were seen in our model of CC in which defibrillation with an automated external defibrillator (AED) within 1 or 2 minutes of VF resulted in successful resuscitation in 100% and 92% of animals, respectively [21]. Only 46% of shocks were successful after 4 minutes, and after 6 minutes survival decreased further to 25% (p<0.0001). The AED recognized VF with a 98% sensitivity and the specificity for non-shockable rhythms was 100%. Based on these data, more widespread access to AEDs at organized youth sporting events and the training of personnel in their early use would likely achieve substantial increase in the survival of CC victims. Indeed, successful resuscitation of victims of CC with AEDs has been observed. However, the importance of developing more effective primary prevention strategies and promoting their widespread use should also not be overlooked. This point is underscored by the recent tragic case reported in a 22-year-old student who suffered a CC event during an intercollegiate lacrosse game [22]. Despite early AED application and prompt defibrillation (within 2 minutes of his collapse), spontaneous circulation could not be restored. The student died an hour later in a neighboring emergency department.

## Conclusion

CC is being reported with increasing frequency and young athletes tend to be most at-risk. Sudden cardiac death results from the instantaneous induction of VF following a precordial chest wall blow. A narrow window of vulnerability corresponding to the time of intrinsic increase in repolarization dispersion, just before the peak of the T wave has been identified. In addition, several other variables, including the impact location and the hardness and velocity of the projectile relate to the production of a critical LV pressure that is necessary to induce VF. Rapid LV pressure rise following chest impact results in myocardial stretch and activation of ion channels, including the K^+^_ATP_ channel, via mechano-electric coupling. Inward current through these mechano-sensitive ion channels results in further augmentation of repolarization and non-uniform myocardial activation, and is the cause of premature ventricular depolarizations that are the triggers of VF. Survival in the USCCR is quite poor and largely dependes on institution of early defibrillation and resuscitation. Increasing the availability of AEDs at organized youth sporting events should enhance the safety of young athletes. In addition, the importance of developing more effective primary prevention strategies for CC - such as effective chest protectors and safety balls - and promoting their widespread use is needed.

## Figures and Tables

**Table 1:**
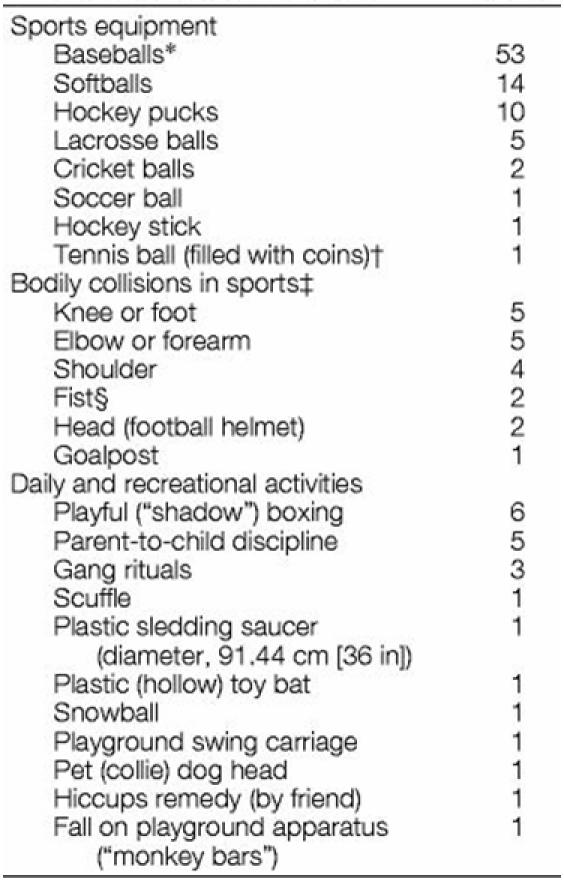
Characteristics of Chest Blows in 128 Commotio Cordis Events

*Includes 4 T-balls. †Training device for baseball pitchers. ‡Baseball, football, karate, soccer, basketball, and hockey. §Closed or open fist (including jab, push, slap). Reprinted with permission from: Maron BJ, Gohman TE, Kyle SB, Estes NA, 3rd, Link MS. Clinical profile and spectrum of commotio cordis. JAMA 2002;287(9):1142-6. Copyright ©  (2002), American Medical Association. All rights reserved.
